# Clinical repercussions of statin use during pregnancy: a review of the literature

**DOI:** 10.61622/rbgo/2025rbgo2

**Published:** 2025-03-17

**Authors:** Joan Lins Serafim, Pedro Lucas Santos de Menezes Teles, Amanda Katharinne Souza Lima, Jéssica dos Santos Coelho, Paloma Luna Maranhão Conrado, Valda Lúcia Moreira Luna, Pauliana Valéria Machado Galvão, George Alessandro Maranhão Conrado

**Affiliations:** 1 Universidade de Pernambuco Recife PE Brazil Universidade de Pernambuco, Recife, PE, Brazil.

**Keywords:** Pregnancy, Pregnant woman, Hydroxymethylglutaryl-CoA reductase inhibitors, Pre-eclampsia, Antihypertensive agents, Pravastatin

## Abstract

Statins are the most widely used pharmacological class for treating hyperlipidemia, although they are contraindicated during pregnancy. This study aims to demonstrate the clinical effects of statins in pregnant women through an interactive review. Fifteen original articles were selected, in English or Portuguese, within of five years. Statins have not been associated with the development of fetal malformations and their use may be useful in preventing unfavorable cardiovascular outcomes, with the potential to reduce oxidative stress and angiogenic dysfunction. However, the use of statins to prevent pre-eclampsia in humans has not been properly clarified and further studies are needed. Pravastatin is considered safer than statins for use during pregnancy.

## Introduction

Statins are the most widely used drugs for the treatment of hyperlipidemia.^(1)^ Their mechanism of action is based on inhibiting the enzyme 3-hydroxy-3-methyl-glutaryl-coenzyme A reductase (HMG-CoA), through their affinity for the enzyme's active site.^(2)^ This inhibition is reversible and competitive with the HMG-CoA substrate, thus decreasing the cholesterol content in the hepatocyte and determining a reduction in circulating levels of low-density lipoproteins (LDL).^(2)^

Although, the safety of these drugs during the gestational period is uncertain. Consequently, the first statin developed, lovastatin, was contraindicated during pregnancy because of its possible relationship with congenital anomalies.^(3)^ Thus, the Food and Drug Administration (FDA), the agency responsible for controlling the safety and efficacy of drugs in the United States, considered that the demonstrated risk of causing birth defects outweighed the benefits of statin use and recommended their discontinuation from conception attempts until the end of breastfeeding.^(4,5)^

However, in July 2021, new studies were analyzed, and the FDA removed the restrictions on statins. From this point of view, it was noted that the previous recommendation was built on experimental studies in animals with higher doses than those used in humans.^(5)^ In addition, more recent studies involving humans have not demonstrated an association of statins with an increased risk of fetal malformations.^(6)^

Current animal studies have also evaluated the use of statins in the prevention of preeclampsia (PE). This multisystemic disease is part of the spectrum of hypertensive disorders of pregnancy, affecting around 5.0 to 8.0% of all pregnancies and being the third cause of maternal mortality in the world.^(7)^ The clinical profile of PE is hypertension and proteinuria after 20 weeks of gestation. Hypertension is evidenced by systolic blood pressure ≥140 mm Hg and/or diastolic blood pressure ≥90 mm Hg, and proteinuria is perceived by urinary excretion of ≥300 mg of protein in a 24-hour sample. In addition, PE can show signs of severity, manifesting as liver damage, hemolysis, increased nitrogenous waste, neurological damage, and hypertensive emergencies. If left untreated, PE can lead to fatal complications for both mother and fetus. Despite its seriousness, the only treatment available for PE is the control of high blood pressure in the mother and, ultimately, the deliverance of the placenta and fetus, with the serious risks associated with neonatal prematurity. Therefore, identifying drugs with the potential for beneficial treatment of PE remains of the greatest importance.^(8)^

It has been verified that statins, specifically pravastatin, can reverse the imbalance in angiogenic and antiangiogenic factors that contribute to cause PE.^(9)^ Moreover, due to their pleiotropic effects, they can act neutralizing the biological mechanisms that result in the phenotype of this pathology.^(10)^

Considering the aforementioned, the effect of statins during pregnancy, although still controversial, can bring benefits to users. Thus, this review aims to compile data on the clinical repercussions of statins use in pregnant women, hoping to facilitate the identification of benefits and the understanding of the mechanisms of a medication that can help prevent maternal and neonatal diseases.

## Methods

**Figure 1:**
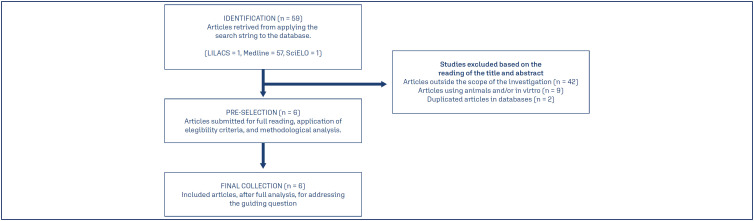
Articles selection process

This is an integrative literature review with the objective of systematizing the results of the search on the specific theme. Through previous literature analysis, the following guiding research question was defined: "What are the possible clinical repercussions of statin use during pregnancy?". Therefore, the methodological process was composed of the following steps: 1. Definition of eligibility criteria; 2. Research strategy design; 3. Data collection and sample delimitation by two reviewers; 4. Critical data analysis, discussion, and synthesis of results by two reviewers. Data collection was performed from August 2022 to February 2023 by consulting the Medical Literature Analysis and Retrieval System Online (MEDLINE), Scientific Electronic Library Online (SciELO), and Latin American and Caribbean Literature on Health Sciences (LILACS) databases. The controlled descriptors were selected from the Health Sciences Descriptors/Medical Subject Headings (DeCS/MeSH) register, in English, and combined by the Boolean operator AND, forming the following search strategy: "Hydroxymethylglutaryl-CoA Reductase Inhibitors" AND "Pregnancy". The eligibility criteria established were original, complete, and freely accessible articles, in English or Portuguese, within a five-year time frame, and presenting a detailed description of the relationship between statin use and pregnancy. Theses, dissertations, review articles, meta-analyses, letters to the editor, reports or case series were not included, and articles repeated in databases, studies outside the scope of the investigation, animal studies, and in vitro studies were excluded. After applying the identification, screening and eligibility criteria process as described in figure 1, six articles relevant to the research question remained in the final collection.

In order to ensure the ethical aspects supporting to this study, the authorship and citations of each publication submitted were formally referenced. Given the fact that this was a literature review that did not directly involve research with human beings, the present work does not require the approval of the Research Ethics Committee, according to Resolution 510/2016 of the Brazilian National Health Council.

## Results

The selected studies presented descriptions of statin use and its pharmacological effects in pregnancy. Data were extracted and processed using a tool designed by the authors with the following fields: authors and year of publication, objective, methodology, and results. The characteristics of each study are presented in chart 1. All selected articles were written in English and compiled data from various countries, such as the United Kingdom, the United States, Taiwan, Spain, and Serbia, as well as one study conducted concurrently in 3 countries (England, Spain, and Belgium)

**Chart 1:** Distribution of articles included in the integrative review

Author(s)	Objective	Methodology	Results
Chang et al.^(3)^	To evaluate the perinatal outcome associated with maternal use of statins during pregnancy.	Retrospective cohort from Taiwan, with selection of 469 women who used statin drugs in pregnancy and 4690 who did not (1:10 matching, considering maternal age and year of birth).	There was no association between anomaly and exposure to statins, however, the use of statins led to risk of spontaneous premature birth, low birth weight, and lower first minute Apgar score.
Ahmed et al.^(11)^	To evaluate the effects of pravastatin on plasma levels of soluble tyrosine kinase-1 (sFlt-1) and angiogenic protein in cases of preeclampsia (PE).	UK randomized clinical trial (RCT) with 62 PE patients between 24 and 32 GTW. 1:1 pairing. The intervention group received 40mg daily of pravastatin while the control received a placebo until the time of delivery. Maternal serum sFlt-1 levels were measured 3 days after randomization, the first 14 days and 6 weeks after delivery.	There was no statistical evidence of the effect of pravastatin in prolonging pregnancy and in relation to plasma levels of sFlt-1 at any of the time points measured. Similarly, there were no serious adverse reactions attributable to treatment with pravastatin.
Costantine et al.^(12)^	To evaluate the safety and pharmacokinetic parameters of pravastatin for women at high risk of PE.	Multicenter RCT with 20 women at high risk for PE between 12 and 16 weeks and prior history. 1:1 pairing, one group with pravastatin 20mg and one with placebo, from study entry until the day of delivery. Pravastatin concentration, PE progression, and tests of angiogenesis-related factors were performed.	PE occurred in 10.0% with pravastatin and 45.0% with placebo and preterm birth in 20.0% and 55.0% respectively. Birth weight and Gestational Age at delivery were higher in the intervention group compared to the control. There was no statistical difference.
Döbert et al.^(13)^	Evaluate the use of pravastatin in women at high risk of full-term PE to reduce the incidence of deliveries with this outcome.	RCT conducted in 3 countries, 1,120 high-risk pregnant women from term PE. 1:1 pairing. One group using 20mg pravastatin or placebo daily from 35 to 37 weeks until 41 GTW, labor or 1 day before cesarean section.	There was no evidence of the effect of pravastatin on reducing PE and the incidence of secondary maternal outcomes.
Jurisic et al.^(14)^	To evaluate the use of pravastatin associated with L-arginine in improving uteroplacental hemodynamics and preventing neonatal adverse effects.	NRC with 10 pregnant women from Serbian hospitals, with uteroplacental dysfunction between 20 and 22 GTWs. The groups were divided equally and the intervention group received a daily supply of 40mg pravastatin and 1.5g L-arginine compared to an untreated control.	In patients treated with pravastatin and L-arginine, pregnancies continued for 119 days compared with 26 days in the untreated group, plus neonates had a mean birth weight of 3,050 g among pregnant women on the medication compared with 644 g in the control group.
Mendoza et al.^(15)^	To analyze the effect of pravastatin on gestational outcome in women with early fetal growth restriction.	NRC with a sample of 38 women seen from Barcelona with single gestation, restricted fetal growth, no fetal disorders and another 19 historical control women matched for gestational and maternal characteristics. The intervention group used 40mg pravastatin daily compared to the untreated control after diagnosis.	There was no statistically significant difference between the groups in time from diagnosis to delivery or fetal birth weight. Placental growth factor soluble sFlt-1 levels were 10.1 in the group taking pravastatin compared with 67.0 in the control group.

sFlt-1 - soluble tyrosine kinase-1; PE - Pre-eclampsia; RCT - Randomized Clinical Trial; GTW - gestational week; NCT - Non-Randomized Clinical Trial

## Discussion

Cholesterol and its derivatives are essential components for fetal development, being involved in the synthesis of steroid hormones and cell membranes, and are also crucial for the development of the peripheral nervous system. In this regard, the possible teratogenicity of statins has been associated with the interruption of cholesterol synthesis.^(3)^

However, one study showed that exposure to statins during pregnancy was not associated with congenital anomalies.^(3)^ Additionally, of the broad spectrum of statins available, pravastatin is believed to be the safest to use during pregnancy, possibly due to its hydrophilic nature.^(16)^ Because it is hydrophilic, pravastatin has great difficulty in crossing the blood-brain barrier, demonstrating greater safety compared to lipophilic statins.^(17)^ Another study found that cells treated with higher doses of simvastatin and rosuvastatin, other statins, experienced loss of elongated cell shape, and the Pravastatin, on the other hand, did not affect cell shape, suggesting it is the safest of the three drugs at the doses used.^(18)^

In the study by Costantine et al.,^(12)^ the pregnant women who used pravastatin had the same rate of congenital anomalies as the pregnant women in the placebo group. Accordingly, the use of pravastatin in women with uteroplacental vascular dysfunction has also demonstrated that it is a safe medication for use during pregnancy.^(14)^

In addition, an increased risk of congenital malformations was not associated in several cohorts of women exposed to pravastatin during pregnancy.^(12)^ It is worth noting that continued use of statins in women on prior use three months into pregnancy did not increase the risk of adverse neonatal outcomes. However, their use should be performed with caution.^(3)^

According to the most accepted theory, the pathophysiology of PE begins with a dysfunction of trophoblastic invasion in the spiral arteries, resulting in insufficient blood perfusion and, consequently, in placental ischemia.^(12)^ From this, increased oxidative stress and release of antiangiogenic factors are observed, promoting a generalized endothelial dysfunction that culminates in increased blood pressure.^(19)^

Mendoza et al.^(15)^ demonstrated that during a pregnancy with PE, concentrations of angiogenic factors, such as placental growth factor (PIGF), decrease already in early pregnancy, while antiangiogenic factors, such as soluble tyrosine kinase-1 (sFlt-1), increase. In parallel, the study by Ahmed et al.^(11)^ showed that a high ratio of sFlt-1:PIGF is associated with increased adverse perinatal outcomes and reduced time to delivery. It was also observed that women with PE who received pravastatin had the lowest baseline sFlt-1:PIGF ratio compared with women who received placebo.^(11)^

Furthermore, in an analysis of isolated uterine microvascular cells, pravastatin decreased the release of other factors associated with endothelial dysfunction, such as endothelin-1 (ET-1), without loss of cell viability.^(18)^

It is also worth noting that nitric oxide (NO), an important vasodilator in vascular adaptations during pregnancy, is decreased in PE, and the increase in its plasma levels is an interesting measure as a therapeutic strategy.^(20)^ In this perspective, it was found that the level of serum NO was significantly elevated in rats with PE treated with pravastatin.^(21)^

In addition, an increase in NO bioavailability and an improvement of NO-dependent vasodilatory mechanisms in the peripheral circulation after the use of pravastatin was verified in chicken embryos.^(16)^ This increased bioavailability can be explained by increased activity of the endothelial nitric oxide synthase enzyme (eNOS).^(20)^

Furthermore, pravastatin can also prevent the elevated oxidative stress induced by hypertension in pregnancy.^(22)^ Complementarily, it inhibited the interleukin-6/protein signal transducer and activator of transcription 3 (IL-6/STAT3) signaling pathway, alleviating oxidative stress and reducing placental trophoblast cell apoptosis in rats with PE.^(21)^

PE is a multisystemic disorder of pregnancy that represents an important cause of perinatal morbidity and increased cardiovascular risk even after delivery.^(23)^ However, currently, there are few prophylactic measures for it. In this perspective, studies in animals, pilot and pre-clinical and clinical trials have evidenced benefit of statins use for correction of factors associated with PE pathogenesis, such as angiogenic dysfunction, endothelial injury, oxidative stress and inflammation.^(12)^

One study demonstrated a reduction in the occurrence of early onset PE, improved uteroplacental hemodynamics and fetal growth when taking pravastatin and arginine.^(14)^ However, there was no evidence of the effect of pravastatin use in reducing delivery with PE after starting the medication at 35 to 37 weeks of gestational age.^(13)^ Another study with earlier initiation of the drug, between 12 and 16 weeks and 6 days gestation, with the same daily pravastatin dose also showed no significant difference in adverse events between the groups.^(12)^

Statins represent the class of drugs commonly used to treat dyslipidemia and reduce the risk of unfavorable cardiovascular outcomes.^(18)^ When maintained in pregnant women previously using such medication, no adverse perinatal outcomes were identified. However, the beginning of statins use during pregnancy was related to low birth weight, higher chance of premature delivery and lower Apgar score in the first minute.^(3)^ This information favors the use of this class of drugs in former users, aiming to reduce the risk of unfavorable cardiovascular events during pregnancy in dyslipidemic women.

Furthermore, statins emerge as a possibility to reduce the cardiovascular risks of PE in the long term. Despite being considered a transient disease of pregnancy, PE has shown association with future chronic hypertension and cardiovascular disease, suggesting that it may represent more than a pathology restricted to pregnancy.^(24)^ The study by Kräker et al.^(23)^ also indicated that pravastatin aided cardiac remodeling and improved cardiac output in rats. In addition, there was a reduction in endothelial dysfunction after pregnancy in the genitor animal.^(24)^

## Conclusion

It appears that the use of statins during pregnancy, especially pravastatin, is not related to fetal malformations. However, in view of possible adverse neonatal outcomes, caution should be exercised. In addition, the use of statins has beneficial maternal effects, reducing the risk of unfavorable cardiovascular events. It is noteworthy that studies in animals have shown, by different mechanisms, benefits from the use of this drug for the prevention of PE. However, the same results were not observed in large studies in humans, evidencing a limitation of the investigation, given the scarcity of studies in humans with clinical evidence for the use of statins in these settings. Thus, the findings increase interest in pravastatin as a potential prophylaxis for PE. However, it is suggested that additional studies be conducted before clinical implementation, aiming to define therapeutic efficacy, the optimal time to start the drug, and the most appropriate dose.

## References

[1] Vahedian-Azimi A, Bianconi V, Makvandi S, Banach M, Mohammadi SM, Pirro M (2021). A systematic review and meta-analysis on the effects of statins on pregnancy outcomes. Atherosclerosis.

[2] Esteve-Valverde E, Ferrer-Oliveras R, Gil-Aliberas N, Baraldès-Farré A, Llurba E, Alijotas-Reig J (2018). Pravastatin for preventing and treating preeclampsia: a systematic review. Obstet Gynecol Surv.

[3] Chang JC, Chen YJ, Chen IC, Lin WS, Chen YM, Lin CH (2021). Perinatal outcomes after statin exposure during pregnancy. JAMA Netw Open.

[4] Reis MA, Macedo GP, Amorim RB, Pereira DA, Medeiros II, Torres KR (2022). Riscos de uso de estatinas na gestação: uma revisão de literatura. Braz J Health Rev.

[5] Bittencourt MS (2022). Statins and pregnancy – new FDA recommendations. Arq Bras Cardiol.

[6] Karadas B, Uysal N, Erol H, Acar S, Koc M, Kaya-Temiz T (2022). Pregnancy outcomes following maternal exposure to statins: a systematic review and meta-analysis. Br J Clin Pharmacol.

[7] Ma'ayeh M, Rood KM, Kniss D, Costantine MM (2020). Novel interventions for the prevention of preeclampsia. Curr Hypertens Rep.

[8] Girardi G (2017). Pravastatin to treat and prevent preeclampsia. Preclinical and clinical studies. J Reprod Immunol.

[9] Gajzlerska-Majewska W, Bomba-Opon DA, Wielgos M (2018). Is pravastatin a milestone in the prevention and treatment of preeclampsia?. J Perinat Med.

[10] Smith DD, Costantine MM (2022). The role of statins in the prevention of preeclampsia. Am J Obstet Gynecol.

[11] Ahmed A, Williams D, Cheed V, Middleton L, Ahmad S, Wang K (2020). Pravastatin for early-onset pre-eclampsia: a randomised, blinded, placebo-controlled trial. BJOG.

[12] Costantine MM, West H, Wisner KL, Caritis S, Clark S, Venkataramanan R (2021). A randomized pilot clinical trial of pravastatin versus placebo in pregnant patients at high risk of preeclampsia. Am J Obstet Gynecol.

[13] Döbert M, Varouxaki AN, Mu AC, Syngelaki A, Ciobanu A, Akolekar R (2021). Pravastatin versus placebo in pregnancies at high risk of term preeclampsia. Circulation.

[14] Jurisic A, Jurisic Z, Lefkou E, Girardi G (2021). Pravastatin plus L-arginine prevents adverse pregnancy outcomes in women with uteroplacental vascular dysfunction. Vascul Pharmacol.

[15] Mendoza M, Ferrer-Oliveras R, Bonacina E, Garcia-Manau P, Rodo C, Carreras E (2021). Evaluating the effect of pravastatin in early-onset fetal growth restriction: a nonrandomized and historically controlled pilot study. Am J Perinatol.

[16] Itani N, Skeffington KL, Beck C, Niu Y, Katzilieris-Petras G, Smith N (2020). Protective effects of pravastatin on the embryonic cardiovascular system during hypoxic development. FASEB J.

[17] Carson RA, Rudine AC, Tally SJ, Franks AL, Frahm KA, Waldman JK (2018). Statins impact primary embryonic mouse neural stem cell survival, cell death, and fate through distinct mechanisms. PloS One.

[18] de Alwis N, Beard S, Mangwiro YT, Binder NK, Kaitu'u-Lino TJ, Brownfoot FC (2020). Pravastatin as the statin of choice for reducing pre-eclampsia-associated endothelial dysfunction. Pregnancy Hypertens.

[19] Rana S, Lemoine E, Granger JP, Karumanchi SA (2019). Preeclampsia: pathophysiology, challenges, and perspectives. Circ Res.

[20] Pánczél Z, Kukor Z, Supák D, Kovács B, Kecskeméti A, Czizel R (2019). Pravastatin induces NO synthesis by enhancing microsomal arginine uptake in healthy and preeclamptic placentas. BMC Pregnancy Childbirth.

[21] Wang GJ, Yang Z, Huai J, Xiang QQ (2020). Pravastatin alleviates oxidative stress and decreases placental trophoblastic cell apoptosis through IL-6/STAT3 signaling pathway in preeclampsia rats. Eur Rev Med Pharmacol Sci.

[22] Chimini JS, Possomato-Vieira JS, da Silva ML, Dias-Junior CA (2019). Placental nitric oxide formation and endothelium-dependent vasodilation underlie pravastatin effects against angiogenic imbalance, hypertension in pregnancy and intrauterine growth restriction. Basic Clin Pharmacol Toxicol.

[23] Kräker K, O’Driscoll JM, Schütte T, Herse F, Patey O, Golic M (2020). Statins reverse postpartum cardiovascular dysfunction in a rat model of preeclampsia. Hypertension.

[24] Garrett N, Pombo J, Umpierrez M, Clark JE, Simmons M, Girardi G (2018). Pravastatin therapy during preeclampsia prevents long-term adverse health effects in mice. JCI Insight.

